# Ethical Issues in Social Media Recruitment for Clinical Studies: Ethical Analysis and Framework

**DOI:** 10.2196/31231

**Published:** 2022-05-03

**Authors:** Bettina M Zimmermann, Theresa Willem, Carl Justus Bredthauer, Alena Buyx

**Affiliations:** 1 Institute of History and Ethics in Medicine, School of Medicine Technical University of Munich Munich Germany; 2 Institute for Biomedical Ethics University of Basel Basel Switzerland; 3 Department of Science, Technology and Society School of Social Sciences and Technology Technical University of Munich Munich Germany

**Keywords:** social media, clinical studies, clinical trials, ethics, recruitment

## Abstract

**Background:**

Social media recruitment for clinical studies holds the promise of being a cost-effective way of attracting traditionally marginalized populations and promoting patient engagement with researchers and a particular study. However, using social media for recruiting clinical study participants also poses a range of ethical issues.

**Objective:**

This study aims to provide a comprehensive overview of the ethical benefits and risks to be considered for social media recruitment in clinical studies and develop practical recommendations on how to implement these considerations.

**Methods:**

On the basis of established principles of clinical ethics and research ethics, we reviewed the conceptual and empirical literature for ethical benefits and challenges related to social media recruitment. From these, we derived a conceptual framework to evaluate the eligibility of social media use for recruitment for a specific clinical study.

**Results:**

We identified three eligibility criteria for social media recruitment for clinical studies: information and consent, risks for target groups, and recruitment effectiveness. These criteria can be used to evaluate the implementation of a social media recruitment strategy at its planning stage. We have discussed the practical implications of these criteria for researchers.

**Conclusions:**

The ethical challenges related to social media recruitment are context sensitive. Therefore, social media recruitment should be planned rigorously, taking into account the target group, the appropriateness of social media as a recruitment channel, and the resources available to execute the strategy.

## Introduction

Effective patient recruitment has been one of the most cited barriers to clinical studies [1,2]. Up to 60% of trials are delayed or canceled because of a lack of enrollment [3-5]. In the recent past, social media recruitment has been successfully used for different clinical studies (eg, smoking cessation [6], type 1 diabetes [7], and HIV [8]), which has raised hopes for the improvement of the research process, quality, and efficiency. The COVID-19 pandemic has further enhanced the use of social media platforms.

However, researchers are calling for a more thorough evaluation of recruitment effectiveness, quality, and cost-effectiveness [9], as well as their ethical, legal, and social implications. Recent publications have raised doubts about whether social media can be used for recruitment purposes while preserving fundamental cornerstones of research ethics and biomedical ethics, as reflected in the US Health Insurance Portability and Accountability Act, the General Data Protection Regulation of the European Union, the Declaration of Helsinki, and the Belmont Report, to name a few examples [3,10-15].

Few scholars have examined the unique ethical, legal, and social issues arising from social media recruitment at a metalevel. Gelinas et al [3] have provided an overview of the ethical issues related to social media recruitment in research. They mainly addressed concerns related to user privacy and investigator transparency and provided practical recommendations for internal review boards and investigators. In particular, they claimed that social media recruitment should follow the same standards as traditional recruitment strategies. In this review, we aim to continue this work but take a broader view of the ethical issues that might arise with regard to social media recruitment.

We explore the uniqueness of social media recruitment compared with more traditional forms of patient recruitment. In doing so, we consider the novel architectures of social media platforms and the possibilities they offer for recruitment communication compared with prior web-based and offline one-to-one and one-to-many communication channels, such as email recruitment or billboard recruitment. Our analysis is based on the benefits and challenges of complex community-based communication opportunities that unfold based on different privacy settings. We also examine the application of machine learning techniques for predictive analytic purposes of user behavior based on central databases [16].

We aim to target researchers involved in clinical studies who are considering incorporating social media into their recruitment strategies. Specifically, we seek to provide a comprehensive overview of the ethical issues to be considered from conceptual and empirical perspectives (including the potential benefits and risks) and provide practical recommendations on how to take these issues into account when using social media as a recruitment tool. Therefore, our recommendations are formulated with a view toward practical application.

## Methods

We conducted a structured, nonsystematic review of the empirical evidence available for social media recruitment and its links to ethical challenges. First, we broadly reviewed the normative and empirical scientific literature on social media recruitment. Then, we identified relevant papers based on their abstracts through searches in interdisciplinary databases (PubMed [MEDLINE], Web of Science, Google Scholar, and the university library catalogs of the Technical University Munich and Ludwig-Maximilian University Munich) using the initial keywords *social media* AND *recruitment* AND clinical trial*. We also screened the references of the relevant articles. From the publications relevant to social media recruitment, we collected all relevant ethical issues and grouped them thematically according to the principles of biomedical ethics [17], namely, autonomy, justice, nonmaleficence, and beneficence. We then checked the ethical issues for conceptual completeness using 2 well-established normative frameworks for research ethics as conceptual guidance [18,19]. These frameworks guide important concepts of state-of-the-art biomedical and ethical research, such as privacy, informed consent, specific protection of the vulnerable, and other potential risks relevant in this context. After identifying conceptually relevant ethical benefits and challenges, we performed additional literature searches to gain more focused insights into the available empirical literature on these benefits and challenges related to social media recruitment for clinical studies. The details of these searches are available in Multimedia Appendix 1.

We present our results in 3 thematic chapters that investigate the benefits of social media recruitment for clinical studies and the associated ethical challenges (part A). As ethical benefits and challenges are highly context sensitive and require a thorough risk-benefit analysis for each clinical study, we used our findings to identify 3 dimensions that should be considered in an ethical assessment of any clinical study considering social media recruitment (part B). In the *Discussion* section, we analyze the practical implications of these dimensions in the context of clinical studies.

## Results

### Part A: Benefits and Challenges of Social Media Recruitment in Clinical Studies

#### Trust, Transparency, and Autonomy

##### Benefit: Promoting Trust, Transparency, and Autonomy

Conceptual and empirical research has encouraged the claim that social media can promote trust, transparency, and autonomy in research studies. For instance, the options for bilateral and multilateral interactions on social media enable participants to learn about the results of the clinical study in which they participate [20,21], which increases trust and transparency [22,23]. This allows for an individually adapted level of engagement between participants and researchers “in an era where the patients are collaborators and there is a continuum of need from paternalism to complete autonomy” [24]. Thus, when intended as an instrument to improve autonomy, social media provides an opportunity to promote patient empowerment [21]. In the realm of patient-led research and citizen science, social media can serve as a platform to bring researchers, patients, and other stakeholders together and foster collaboration [25,26]. This includes researchers being transparent about what data are collected and asking for feedback on the study results, thereby encouraging patient engagement [27]. As health data become increasingly accessible to individual patients outside the clinical setting, this is particularly important. However, data-rich medicine also gives rise to challenges for health care professionals and patients alike, such as supporting digital data practices or contextualizing them meaningfully [28]. In-depth and continuous exchanges between participants and researchers are needed to promote trust, transparency, and autonomy, which could be included in a study’s recruitment process.

Recruitment process reports suggest that using social media improves autonomous decision-making with regard to study participation by offering the possibility of multiple contact points over time, which reduces time pressure and supports informed decision-making [23,29]. Moreover, information can be presented multimodally (visual, aural, or tactile), which could improve the understanding of study-related information [30]. Reports of users sharing study-related content on their social media accounts, as well as analyses of responses and reactions on social media, have empirically demonstrated how perceived trust and transparency can be fostered on social media [31,32]. However, there are several ethical challenges, including issues related to the tension between information, nudging, and persuasion (refer to the *Challenge: Information, Persuasion, and Nudging* section), informed consent issues (refer to the *Challenge: Informed Consent* section), privacy and data security issues (refer to the *Challenge: Privacy and Data Security* section), and low digital literacy (refer to the *Challenge: Digital Literacy* section).

##### Challenge: Information, Persuasion, and Nudging

The boundaries between information and persuasion, as well as between education and advertisements, are ambiguous. Information processing has a direct effect on cognitive and emotional responses, motivation, and persuasion to action [33]. The concept of nudging people into beneficial behaviors [34] provides a potential framework that ethically justifies a certain level of persuasion, as long as it serves the best interest of the person. The nudging concept has been discussed extensively—and controversially—in the context of public health [35-38], informed consent in the clinic [39,40], and when providing incentives for research participation. More recently, it has also been discussed in terms of promoting privacy-preserving behavior in social media users [41-43]. Although the definition and scope of nudges are subject to debate, we refer to nudging as used by VanEpps et al [44], who suggested 3 forms of interventions to nudge research participation. The first form is simply *providing information* about the study, which is disadvantageous as it might lead to a lower recruitment accrual than other forms of nudging. Moreover, information can be provided in more or less persuasive ways, and what is perceived as appropriate information provision is highly context dependent. In any event, transparency regarding social media–related activities and interventions is an important prerequisite for any social media activity related to a clinical study [15] (see also *Challenge: Informed Consent* section).

The second form of nudging is *choice architecture.* For example, default choices might increase recruitment effectiveness but lead to informed consent issues that are unacceptable in the context of clinical studies. However, a recent study among surrogate decision-makers in an intensive care unit setting found no statistical difference between the 2 offers of choice architecture or any evidence of undue and unjust inducements [45].

The third form of nudging includes *monetary or other incentives.* This might lead to undue or unjust inducement and needs to be assessed in a context-specific manner [46,47]. Social nudging in the form of rewarding goal attainment has shown positive effects on individuals’ willingness to act in a group’s interest in the context of vaccine uptake [48]. However, empirical evidence concerning the effectiveness of these forms of nudging in the context of clinical study recruitment is limited and requires further investigation.

There has been little discussion concerning the implications of nudging and ethical assessments between informing users of the existence of a clinical study and persuading them to participate through monetary incentives or persuasive language. This needs further evaluation, as social media recruitment for clinical studies potentially combines impersonal communication methods with individual decision-making for medical interventions.

##### Challenge: Informed Consent

In the context of social media recruitment, informed consent processes must consider (1) when consent is required, (2) in what instances digital consent is sufficient, and (3) what educational and administrative hurdles are necessary to ensure that consent is informed [49].

Regarding the *requirement of consent*, Gelinas et al [3] called for specific consent when a participant’s social network was used for further recruitment, as such methods might reveal private health-related information to a participant’s social network. This consent acknowledges the context-specific adjustment of the informed consent process in different types of social media recruitment activities.

Regarding *digital consent*, empirical studies have suggested that web-based consent is potentially problematic as users are already used to agreeing to terms and conditions without informing themselves about the details [50]. Moreover, the informed consent processes that are applied vary depending on the study design. In web-based survey studies, consent is obtained directly on the web after the user is redirected from the social media platform to an external survey or intervention website [51,52]. In contrast, in randomized controlled trials, consent is typically sought offline after checking the eligibility criteria and before study enrollment [53,54]. Thus, current informed consent practices in medical studies using social media recruitment focus on consent *after* initial recruitment on social media. However, several authors have lamented the lack of guidelines concerning informed consent processes during or before recruitment for clinical trials via social media [3,55].

Regarding *educational and administrative hurdles*, the effectiveness of electronic forms of consent has been compared empirically with traditional paper-based forms. No universal best practices for e-consent have emerged [56,57]. Moreover, although previous studies have compared different forms of consent [58,59], there is a lack of studies that empirically examine the consent process in the context of social media recruitment for clinical studies.

##### Challenge: Privacy and Data Security

An extensive body of literature has investigated the complex and multifaceted concept of privacy; however, none of the existing definitions are universally applicable [60,61]. Here, we refer to the concept of *information privacy*, which is understood as a subset of privacy in general (refer to the studies by Smith et al [60] and Bélanger and Crossler [62] for comprehensive reviews on information privacy). This paper follows the definition of privacy proposed by Bélanger and Crossler [62]: the “desire of individuals to control or have some influence over data about themselves.” Social media recruitment might conflict with this desire, for instance, when researchers or other potential participants tag individuals on social media recruitment posts without their permission and thereby unwillingly link them to a specific study or disease on a public platform [3,63]. Most importantly, recruiting researchers and other social media users may unknowingly cause such privacy violations (see the *Challenge: Digital Literacy* section). As with any other form of data collection, the researcher is responsible for avoiding the disclosure or loss of information collected on social media in connection with a clinical study [15]. However, researchers do not have control over the data shared on these platforms, making data management potentially challenging [49].

Many social media platforms offer features that advertise to a defined target population and use inaccessible algorithms to select them. For instance, Facebook’s proprietary algorithm uses machine learning to infer the advertisements that should be displayed based on users’ previous behavior [64,65]. A recent study showed that feeding machine learning algorithms with user data risks unforeseeable correlations that might be misused for predictive analytics [66]. Mühlhoff [67] argued that these algorithms have the potential to not only disclose details about a user’s future behavior but also estimate details that users have not disclosed about themselves based on combinations of known data points (eg, stated preferences, demographics, and relations with other users). Thus, a social media platform can learn potentially sensitive information that a user does not want to disclose, posing a privacy challenge. Regulations for these procedures are lacking [68], as is empirical evidence of the ethical and social implications of these potentially problematic privacy issues.

##### Challenge: Digital Literacy

Another challenge affecting social media’s potential to improve autonomy is the lack of digital literacy among social media users, defined as the skills and resources that users need to successfully navigate digital environments [69-71]. Individuals with low digital literacy might be at a greater risk of stigmatization or involuntary violation of their own or others’ privacy. Furthermore, low digital literacy, often in combination with a lack of engagement on social media, may prevent individuals from finding and participating in clinical studies that mostly or solely use social media recruitment [31,71,72]. This may cause ethical challenges regarding the equality of access to clinical studies (see the *Challenge: Equality of Access* section). However, the importance of this issue depends on the population being targeted, as a younger population might be best reached via social media.

Empirical evidence suggests that many social media users lack the skills to self-assess potential risks and harms connected to their social media activities, especially when related to their health status [73-75]. Surveys with health and information technology professionals have found that low digital literacy is commonly perceived as causing ethical challenges when social media is used in the context of participatory health applications. Important concerns include accidental sharing of sensitive data and a limited understanding of what the data on social media are used for [76] (see the *Challenge: Information, Persuasion, and Nudging* section).

#### Justice and Nonmaleficence

##### Benefit: Including Marginalized Groups by Accessing Hard-to-Reach Populations

Ethnic minorities and other marginalized groups are often underrepresented in clinical studies, which causes the benefits of medical research to be unequally distributed [77]. This raises important ethical issues concerning discrimination and equitable access to care. First, there are significant differences in the reactions to medical procedures between subgroups of a population, and the underrepresentation of certain groups in clinical studies may lead to negative consequences for the safety and efficacy evidence in underrepresented ethnic groups [78]. Second, participation in clinical studies might also lead to better medical outcomes for study participants [79]. Third, the unwarranted exclusion of minorities from clinical studies represents a form of epistemic injustice—excluding certain populations from generating knowledge may lead to a biased, ungeneralizable body of knowledge [80].

A reason for the unequal representation in clinical studies is the difficulty in reaching certain groups. Researchers are usually aware of problems related to unequal representation and often cite issues when recruiting certain subgroups within a target group [81]. These hard-to-reach populations are often from socially disadvantaged groups, such as those with low socioeconomic status, ethnic minorities, or older adults [2,52,82].

Social media may help tackle these issues by increasing the ability of researchers to recruit hard-to-reach target populations. Owing to the potential of social media to alleviate the problems of unequal representation in clinical studies, Caplan and Friesen [81] even discussed a “duty to tweet.” They stated that researchers should reduce inequalities in clinical studies and posited that targeted social media recruitment might be a valuable tool to meet this obligation.

Empirical assessments suggest that social media recruitment is effective in recruiting certain populations that are difficult to reach using traditional methods. Specifically, studies have shown that social media recruitment can overcome linguistic and educational barriers and reach immigrants and low-education and low-income populations [83,84]. In addition, social media has been useful in recruiting low-prevalence populations [85-87] and young individuals [15,88]. Moreover, social media recruitment has been successfully used to target gay couples for HIV and hepatitis B and C interventions [32,89]. As these considerations are target group–specific, they must be assessed separately for each study.

Social media offers researchers and participants a low-threshold opportunity to engage with research. This is relevant from the perspective of marginalized groups as it provides value and allows for low-threshold access to potentially beneficial care [79].

##### Challenge: Stigmatization of Vulnerable Populations

Although vulnerability originally referred to the limited ability of persons to give informed consent (Department of Health, Education, and Welfare and National Commission for the Protection of Human Subjects of Biomedical and Behavioral Research), the term was recently extended to include a more general definition and was applied to all those “incapable of protecting their own interest” [19]. Although the first definition tends to define vulnerability too narrowly, the second tends to define vulnerability too broadly, such that all human beings could be assigned to one vulnerable group or another [90]. To avoid this, vulnerability (meaning the potential to cause disproportional wrong to potential research participants [91]) should be assessed on a case-by-case basis when planning a research study. Furthermore, uncovered vulnerabilities should be re-examined situationally, as not every individual with a certain characteristic is vulnerable to the same extent under the same circumstances. This includes planning ahead for a patient’s potential situations of vulnerability [92-95]. Clinical studies targeting patients with characteristics that render them vulnerable must consider thorough informed consent processes and options to withdraw consent (refer to the *Challenge: Informed Consent* section). Consequently, both the vulnerability status and informed consent process are highly dependent on the respective groups targeted for recruitment.

Social media recruitment occurs in a public or semipublic sphere [63], which means that the distinction between private and public communication is not always as transparent as in traditional social settings. For instance, social media users might have their own perceptions of private and public spaces on social media [96]. Concurrently, users may not be aware of what purposes their communication is used for or even who can access the content they publish [63,97] (see the *Challenge: Digital Literacy* section). This potentially leads to private information being disclosed involuntarily, whereby users may unwillingly grant access to information on their health status to others or are unable to anticipate the potential consequences of publicly disclosed health-related information (see the *Challenge: Privacy and Data Security* section). In such situations, social media recruitment could lead to stigmatization [3,98]. Fear of stigmatization can be a significant barrier to participation in clinical studies [77] but occur only if individuals are identifiable from disease-related activities, groups, or comments [3]. The risk of stigmatization is closely related to privacy issues [99] and violates the principle of nonmaleficence [17,100]. As marginalized groups are at particular risk of stigmatization on social media, this stands in sharp contrast to the benefit of reaching marginalized groups, as outlined in the *Benefit: Including Marginalized Groups by Accessing Hard-to-Reach Populations* section.

Empirical studies have suggested that stigmatized health conditions are treated differently from nonstigmatized conditions on social media, and individuals with stigmatized diseases prefer anonymity when discussing their condition on the web [101-103]. In general, as Boudewyns et al [104] have shown using examples of sexually transmitted diseases, people talk less about the health conditions to which they attach a stigma. In this respect, interaction with clinical studies in the context of social media recruitment might involuntarily lift this anonymity. Conversely, other findings have suggested that patients with stigmatized conditions turn to social media to build relationships with other patients, acquire new information, and receive emotional support [105-108].

##### Challenge: Equality of Access

Although social media makes certain traditionally hard-to-reach populations more accessible for clinical study recruitment, it might also lower the chances of certain participants accessing the clinical study, particularly those underrepresented on social media. Consequently, the recruitment strategy must consider potential access issues, particularly when recruiting from closed groups [15]. Various empirical evidence suggests that despite the increasing spread of mobile devices [109,110], some groups are less likely than others to engage with technological devices and, therefore, have less access to social media. First, this may be relevant to older adults, as older age is associated with poor digital literacy [111-113] and lower internet use [114]. Second, individuals with lower educational attainment appear to be underrepresented on social media [15,115]. Moreover, low socioeconomic status tends to correlate with low eHealth literacy [116], and individuals with a low socioeconomic status tend to use the internet in *more general and superficial ways* [117,118] (see also the *Challenge: Digital Literacy* section). Thus, recruitment via social media may be challenging when these groups are targeted.

Although the ability of many social media platforms to direct study advertisements toward defined target groups can be useful in addressing unknown patients, it is unclear how the underlying algorithms choose members of the target group [13,119]. Although researchers choose certain patient parameters such as age or residential area, Facebook also uses its activity logs to improve the target algorithm [120]. Hence, researchers have only limited control over who is getting the advertisement and whether this remains stable over time. Consequently, such algorithms not only represent an ethical challenge related to privacy (see the *Challenge: Privacy and Data Security* section) but also make it difficult for researchers to control for the equitable distribution of information and advertisements, which provides challenges related to research quality (see the *Challenge: Research Quality* section). The magnitude of this issue depends on the social media platform and the strategy used for recruitment and, therefore, needs a context-specific assessment.

#### Beneficence

##### Benefit: Increasing Effectiveness and Cost-effectiveness of Recruitment

For ethical reasons, recruitment for clinical studies should be designed as effectively and cost-effectively as possible. Furthermore, choosing the most cost-effective recruitment option is key to achieving the maximum benefit from a given research budget. Early termination of studies because of a lack of participants puts existing study participants through pointless risks and could impair their trust in the research [121]. However, the lack of effective recruitment remains a persistent challenge in clinical studies.

According to some estimates, up to 60% of clinical trials are delayed or canceled because of a lack of enrollment [3-5]. However, social media recruitment strategies have shown early signs of effectiveness in various types of clinical trials [3], including studies of HIV vaccines [8], smoking cessation studies [6], studies of bothersome vulvovaginal symptoms [122], studies targeting patients with type 1 diabetes [7], occipital nerve studies [123], and studies of depression prevention [124]. A systematic review also reported shorter recruitment periods than traditional strategies in health research [88]. However, as success stories are typically published more often than failures, it is expected that the actual effectiveness depends on the target group and recruitment strategy.

Approximately half of the studies that recruited participants for medical research reported that social media is more cost-effective than traditional methods [9]. In a recent review, Brøgger-Mikkelsen et al [125] found that the median cost per enrollee for web-based recruitment strategies was US $72, and the median cost per enrollee for offline recruitment strategies was US $199, with 31% (4/13) of the included studies reporting web-based recruitment to be less cost-effective than offline recruitment. However, clinical trials provide a specific context as clinicians routinely come into contact with potential participants, and inclusion criteria are usually complex and involve clinical data [126,127].

##### Challenge: Research Quality

Existing evidence for recruitment effectiveness and cost-effectiveness of social media recruitment suggests that success is context dependent. The reasons for low recruitment accrual include the overall presentation of the study to the participant possibly being inappropriate or that the chosen platform does not adequately cover the demographic profile of the target population [49]. These issues, along with opaque social media algorithms (refer to the *Challenge: Equality of Access* section) and a digital literacy divide (refer to the *Challenge: Digital Literacy* section), could lead to the low statistical representativeness of those recruited via social media [15,109]. Overrepresented demographics typically include higher education, young age, and lack of immigration history [6,9,128], leading to ethnically homogeneous samples [129]. Although such issues of representativeness are a major concern for population-based studies, whether such issues are applicable depends on the target population of the clinical study. However, social media recruitment does not necessarily lead to effective recruitment results from either a qualitative or a quantitative perspective.

### Part B: Eligibility Criteria of Ethical Social Media Recruitment

#### Overview

**Figure 1:**
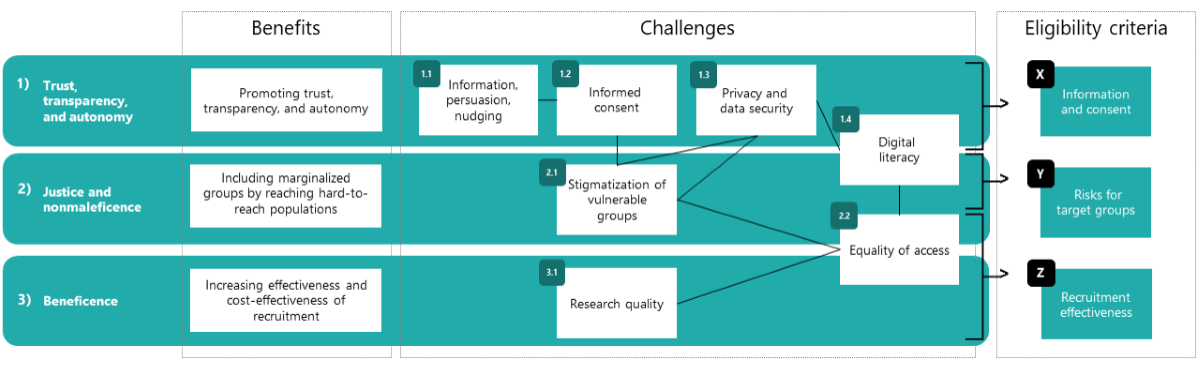
Overview of the benefits and challenges of social media recruitment (part A) and the eligibility criteria to be used for context-specific assessments of social media recruitment strategies.

By reviewing the conceptual and empirical scientific literature, we identified and analyzed the benefits and challenges of social media recruitment for clinical studies. We found that the ethical and practical eligibility of social media recruitment needs to be assessed separately for each study, as ethical benefits and challenges are highly context sensitive. On the basis of the review of ethical issues (part A), we propose three criteria to assess the ethical and practical eligibility of social media recruitment (see Figure 1 for an overview): criterion X, *Information and Consent*; criterion Y, *Risks for Target Groups*; and criterion Z, *Recruitment Effectiveness*. In the following sections, we present the practical implications of each of these criteria and provide an example of how these criteria can be visualized.

#### Criterion X: Information and Consent

##### Transparency

Several forms of transparency must be met in the context of clinical study recruitment on social media to address potential issues related to nudging, informed consent, and privacy.

*Investigator transparency* means that researchers make their identity as researchers visible when interacting with users on social media regarding a study [3].

*Data transparency* includes the disclosure of collected, stored, and used data in study-related social media activities. A major challenge is the nontransparent data processing on most social media platforms, which includes black box machine learning models to predict future behavior [64,65,67]. Open-source social media platforms are currently not widespread and therefore might not be suitable as an effective recruitment strategy. Consequently, researchers often rely on private sector tools for recruitment via social media, where they do not have complete control or are not aware of the potential use of the data collected during the recruitment process. At the very least, this should be acknowledged, and social media users should be made aware of it, particularly in the context of clinical studies.

*Information transparency* is necessary to obtain informed consent. As social media recruitment includes unique issues related to privacy and stigma (refer to the *Challenge: Privacy and Data Security* and *Challenge: Stigmatization of the Vulnerable* sections; also refer to the study by Gelinas et al [3]) that differ from ethical issues arising from the clinical study itself, we propose to distinguish between consent for social media recruitment and traditional patient consent. For social media recruitment, informed consent would depend on how the participants are contacted. Platforms such as Facebook offer several ways to access potential participants [130], such as paid advertisements, where target groups can be specified based on demographic characteristics, interests, and previous web-based activities; project-specific pages, where information about the study is posted on Facebook pages of existing groups related to the topic of interest; and by directly contacting potential participants via private messages [131]. In the following sections, we expand on the questions of whether it is necessary to obtain separate consent for social media recruitment and whether, and to what extent, researchers are responsible for increasing the digital literacy of social media users.

##### Is It Necessary to Obtain Informed Consent for the Recruitment Process on Social Media?

We argue that this depends on the recruitment strategy. Table 1 summarizes what we consider the 4 types of recruitment strategies that have implications for information provision and consent. For the type A and type B strategies, no separate consent is needed as no data are collected on the social media platform. However, it might be necessary to supply information about the study with a disclaimer alerting people of the potential implications concerning their privacy if they share information about the study with others. In contrast, type C and type D strategies include direct contact between researchers or research-related social media channels and (potential) research participants. Although this holds advantages in terms of patient engagement and trust building, it also opens up other issues as study-related data are collected on social media platforms. Participants should be informed about how these data will be used in the context of the study, with whom it will be shared, and how and when it will be deleted, and they should give explicit consent at the beginning of the interaction. The same applies if potential participants are actively recruited in private groups, where they expect to be in a private environment (eg, closed groups on Facebook). Some forms of consent, such as through the group moderator, for contacting these individuals on social media is an ethical imperative in these instances [3]. In type D recruitment strategies, researchers use participants’ social networks to identify and actively address other potential research participants. Although this issue and its implications for privacy and consent have been discussed at length by Gelinas et al [3], we would like to add to their argument that such recruitment strategies should be avoided in particularly vulnerable target populations because of the increased risk of stigmatization, harmful privacy violations, and other psychosocial side effects [98,99].

**Table 1:** SMR^a^ strategies and their implications for information and transparency.

SMR strategy type	Aim	Methods	Characteristics	Scope of targeted audience	Implications for informed consent
Type A	Raise awareness for the study	Advertisements and posts	No engagement in the study on social media	Targeting a broad audience	No signed consent needed
Type B	Actively include social media users in recruiting participants	Sharing of posts, advertisements, and informative material by users	Users are encouraged to reveal connections to a clinical study	Targeting a broad audience	No signed consent is needed, but a disclaimer raising awareness of disclosing connection to the study is required
Type C	Using closed groups for recruitment and community management	Dialogs between researchers and users or in between users; postings in private groups	Study-related data are collected on social media	Targeting a narrow audience	Explicit consent required
Type D	Using the user’s social networks to identify potential participants	Private messaging; active network research of users	Potential user information is revealed to others; user data collected for the study	Targeting a narrow audience	Explicit consent required; caution with vulnerable groups

^a^SMR: social media recruitment.

##### Are Researchers Recruiting for Clinical Studies on Social Media Responsible for Improving the Digital Literacy of Social Media Users?

**Figure 2:**
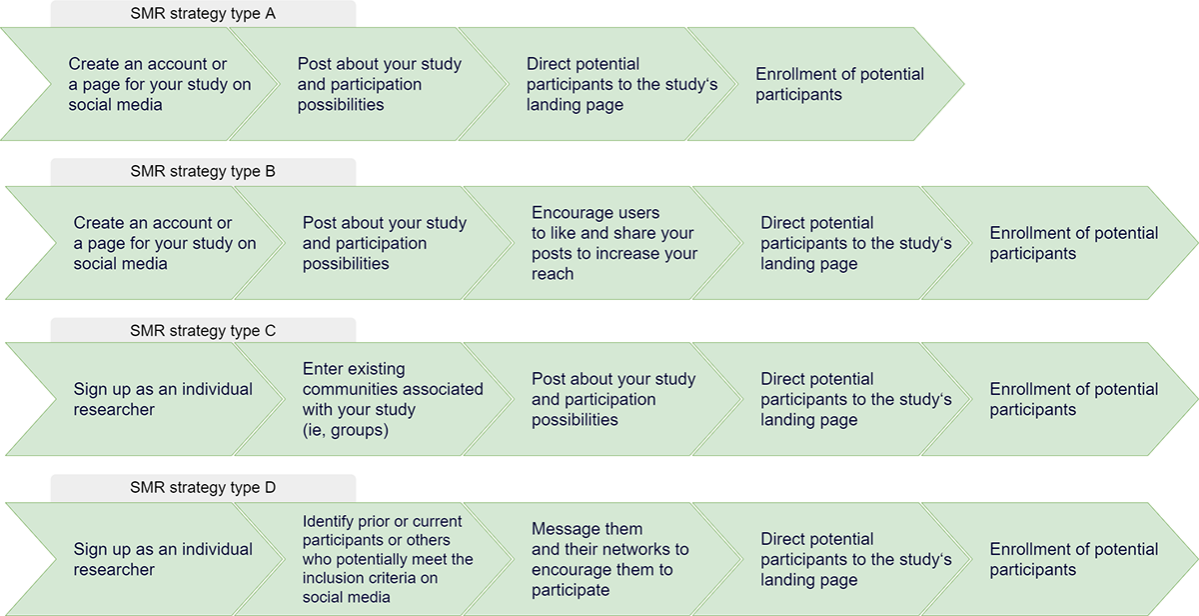
Mock recruitment strategies to illustrate recruitment types A to D. SMR: social media recruitment.

A lack of digital literacy (refer to the *Challenge: Digital Literacy* section) is connected to other ethical challenges, including privacy and stigmatization issues and inadequate patient information [31,71,72,109,110]. On the basis of previous findings [73-76], researchers engaging in social media–based recruitment for clinical studies cannot assume that potential participants are aware of these issues. Researchers are responsible for providing sufficient information to ensure that potential participants do not harm themselves because of a lack of knowledge or awareness. In practice, when using social media recruitment only to raise awareness of the existence of a study (type A or B; Table 1), a small disclaimer may be sufficient. For community engagement in the context of a clinical study (type C or D; Table 1), we recommend developing codes of conduct in community groups and information materials in the form of quizzes or small videos. Such materials should include information on how to protect other patients and provide awareness of the potential risks (including privacy violations and stigma) attached to social media use. Figure 2 illustrates the practical differences of these recruitment types.

#### Criterion Y: Risks for Target Groups

##### Overview

As outlined previously, it appears that both using social media and *not* using it could potentially lead to discrimination and stigmatization. Therefore, the vulnerabilities of target groups should be carefully assessed for web-based and offline recruitment. Considering the broad definition of vulnerability as the incapability to protect one’s interests [19], we propose that the digital literacy of the target group and its risk to attract social stigma as well as the social media recruitment type should be taken into account.

##### Digital Literacy of the Target Group

The literature indicates that insufficient digital literacy is associated with low socioeconomic and educational status and older age [116]. Therefore, target groups meeting one or several of these criteria should be considered vulnerable to social media recruitment. They may be unable to access clinical studies recruiting only on the web and be more prone to misunderstanding the context of clinical studies in the web-based environment. In addition, young target groups tend to underestimate privacy issues on social media and should therefore be contacted with particular care [74,75].

##### Social Stigma of Disease or Other Characteristics

Diseases or characteristics with a social or structural stigma attached to them are varied and can depend on the sociocultural context. These may include characteristics related to sexual orientation [132,133], sexually transmitted diseases [134-136], psychiatric disorders [137,138], or skin diseases [139,140]. Target groups should be carefully evaluated in terms of stigmatized characteristics (see Multimedia Appendix 2 for details), and protective privacy measures should be intensified accordingly. For example, if social media recruitment leads to the public outing of individuals experiencing stigmatized diseases, privacy is particularly important.

##### Recruitment Type

Recruiting from existing participant networks (type D strategies; Table 1) holds particular risks for target groups and should only be considered after explicit consent is given and if the target group is not considered vulnerable, as outlined previously.

#### Criterion Z: Recruitment Effectiveness

##### Overview

To ensure good research quality and effectiveness and avoid unintended harm, researchers should avoid adding social media recruitment as an explorative and inexpensive alternative to other recruitment methods without a detailed implementation plan. Instead, the added value of using social media should be critically examined in the context of a specific clinical study, and a recruitment strategy should be planned a priori when applying for funding. Several dimensions should be considered.

##### Target Group Definition

When considering social media for clinical study recruitment, the age and socioeconomic distribution of the target population must be considered to ensure research quality and improve equality of access. Social media recruitment should only be used if the target group is available on these platforms.

##### Platform Choice

Depending on the technical features, user numbers, user groups, policies, prices, and other characteristics, some social media platforms might be more appropriate for clinical study recruitment than others. These can be used differently by the recruiter by building upon the different features offered by the platforms. For instance, age distribution and other user characteristics vary considerably across platforms [7,49,115]. Therefore, it might be helpful to use multi-platform approaches and triangulate them with other recruitment strategies to avoid inequalities stemming from populations that are inactive on social media or those with low digital literacy [131].

##### Anticipating Patient Responses

Depending on the study design, inclusion criteria, target groups, and recruitment strategy, responses of potential participants will be more or less numerous and accurate in terms of eligibility criteria. For example, type A and type B recruitment strategies target a broader audience, and more (and nonspecific) responses might be expected than in type C and type D strategies (Table 1). The extent of patient responses must be aligned with the resources available to respond to them, particularly when targeting patients with chronic or severe diseases and/or very specific inclusion criteria. For these patients, it might be harmful if they were placing hope in a study they had heard about on social media but then never received a response. However, it might be overwhelming and frustrating for the personnel responsible for patient recruitment if many of the responding patients are not eligible for the study or if resources are not sufficient to respond to all requests. The extent of this problem depends on the type of clinical study.

##### Expertise Within the Research Team

High-quality planning and execution of social media recruitment require specialized knowledge from the research team. Therefore, the clinical study staff should receive formal training, and dedicated recruiters for social media should be employed. Interdisciplinary skills such as platform-specific expertise in terms of use, science communication, and illustrative skills should be required and either represented in the research team or provided through an external provider.

#### The Eligibility Matrix

Depending on the context of a clinical study, the 3 eligibility criteria might have different weights for the overall assessment of social media recruitment. We suggest weighing the risks for target groups (criterion Y) as particularly high as this criterion directly corresponds to the principle of nonmaleficence [141,142]. This criterion usually cannot be improved by an adapted social media recruitment strategy, as it depends on the study-specific target population. In contrast, the other two criteria (X and Z) can be addressed in the recruitment strategy.

**Figure 3:**
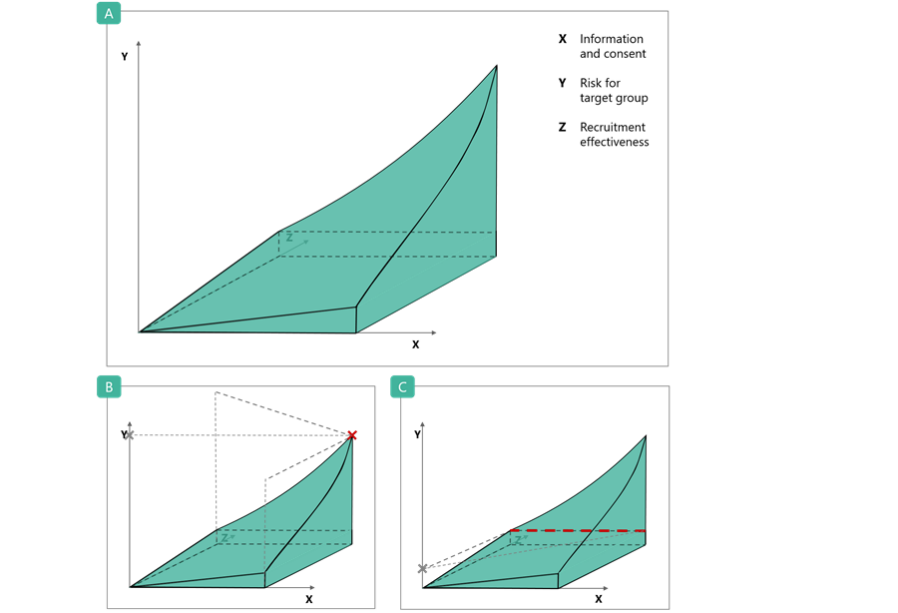
(A) Eligibility matrix for the assessment of social media recruitment for clinical studies. If the result of an assessment of the 3 dimensions occurs within the green volume, social media recruitment can be considered ethical for a particular study. Suitability for using social media for recruitment increases with the respective increase in dimensions X and Z (X: informed consent and Z: recruitment effectiveness) and decreases with a rising risk for the target group (Y: risk for target group). (B) Dashed lines indicate an example of a high-risk target group. X indicates a very limited scope for social media recruitment. (C) Dashed lines indicate an example of a low-risk target group. Dashes represent the scope of the informed consent procedure.

To visualize how the 3 eligibility criteria depend on each other, we arranged them in a 3D matrix (Figures 3A-3C). In cases where there are substantial risks for the target group, social media recruitment would only be permissible in very specific cases (eg, if it is not possible to recruit in any other way or if alternative recruitment methods pose even greater risks). Figures 3B and 3C present 2 examples of these dependencies.

First, if the estimated risk for the target group (y-axis) is particularly high, the estimated recruitment effectiveness (z-axis) also needs to be high, and the informed consent procedure (x-axis) must be extensive for social media recruitment to be considered in a study (Figure 3B). For instance, if we want to recruit participants who have been diagnosed with a stigmatized disease, such as HIV or hepatitis B, the recruitment risks for the target population (Y) are substantial (refer to the *Challenge: Stigmatization of the Vulnerable* section). In this case, social media recruitment must be both effective (X) and have a thoroughly informed consent procedure (Z) to serve as an appropriate recruitment method.

Second, if the overall risk for the target population (y-axis) is estimated to be low and the estimated effectiveness (z-axis) is very high, the range for an acceptable informed consent procedure (x-axis) would broaden (Figure 3C). For example, if we aim to recruit for a clinical study focusing on common health-related behaviors such as vitamin intake and exercise or menopausal symptoms, the risks of recruitment (Y) are lower for the target population. If we expect moderate to good effectiveness in recruiting through social media (X) as we are, for instance, explicitly interested in young participants, the informed consent procedure (Z) only needs to meet the minimum requirements to be adequate.

## Discussion

### Principal Findings

Social media recruitment can offer important benefits for clinical studies, including reaching hard-to-reach populations; increasing recruitment effectiveness; and supporting trust, transparency, and autonomy. These potential advantages make it an ethical imperative to consider this recruitment strategy as a supplement to traditional strategies. However, potential risks strongly depend on the study design, target population, and details of the recruitment strategy. Therefore, each strategy should take into account the target population and the potential risks they might face and should be approved by an institutional review board or ethics committee. In the context of clinical studies, it might be worthwhile to limit social media contact with patients to avoid enhancing the risks of privacy violations or stigmatization and for researchers to create a thorough risk assessment and details on how the recruitment strategy will take these risks into account. However, as social media interactions also potentially increase trust, transparency, and participant engagement, these risks should be weighted context specifically.

If a clinical study targets a particularly vulnerable population, a solution could be to target multiplicators via social media, such as social workers or general practitioners, who then inform the eligible patients about the study. This would lower the risk of privacy violations and stigmatization in vulnerable groups and provide a more beneficial risk-benefit assessment. A potential disadvantage of this approach concerns the indirect steps taken toward recruitment; that is, success depends on the activity of the multiplicator. However, in clinical studies, patients have to come into direct contact with the health care system, and the recruitment process is not as straightforward as it would be for web-based studies.

We identified several research gaps related to the potentially trust-building features of social media recruitment through active engagement, informed consent on the platform itself, and risks of aggravated stigmatization. Relatedly, scholars have identified a lack of ethical and regulatory guidelines, as well as missing reporting standards, that ensure transparency related to social media recruitment for clinical studies [143]. Collecting empirical evidence on the perceptions of researchers, users, and patients concerning social media recruitment for clinical studies is a prerequisite for developing such guidelines. Furthermore, it should be acknowledged that such guidelines are best applied as context specific, as existing privacy and data protection regulations differ between different regions of the world.

Some large and well-known social media platforms (eg, Facebook and Twitter) are more frequently used for clinical study recruitment than others. Thus, they are better documented in empirical studies. As our analysis was partly based on empirical cases of clinical studies that used social media platforms as recruitment tools, other social media platforms might have additional ethical implications for recruitment that were not covered in this contribution.

### Conclusions

Ethical challenges related to social media recruitment are context sensitive. We suggest that the most important challenges for social media recruitment can be assessed by evaluating three dimensions: the level of information and consent, risks for target groups, and effectiveness of the recruitment strategy. These dimensions are interconnected and should be evaluated strategically, critically, and repeatedly. In Multimedia Appendix 2, we provide a checklist with practical recommendations for clinical researchers considering social media recruitment.

Social media recruitment for clinical studies is becoming increasingly common and should only be approved and executed if planned and assessed appropriately. This is particularly important in clinical studies, which might come with additional ethical implications. We suggest that researchers designing a clinical study should use the matrix we have presented to assess a priori whether they should use social media for recruitment.

## Appendix

Multimedia Appendix 1 Search algorithms for literature review.

Multimedia Appendix 2 Checklist for researchers considering social media recruitment for clinical studies.
