# Correction: Ethical Issues in Social Media Recruitment for Clinical Studies: Ethical Analysis and Framework

**DOI:** 10.2196/40848

**Published:** 2022-09-07

**Authors:** Bettina M Zimmermann, Theresa Willem, Carl Justus Bredthauer, Alena Buyx

**Affiliations:** 1 Institute of History and Ethics in Medicine School of Medicine Technical University of Munich Munich Germany; 2 Institute for Biomedical Ethics University of Basel Basel Switzerland; 3 Department of Science, Technology and Society School of Social Sciences and Technology Technical University of Munich Munich Germany

In “Ethical Issues in Social Media Recruitment for Clinical Studies: Ethical Analysis and Framework” (JMIR 2022;24(5):e31231) the authors made one addition to the Acknowledgments section.

The funding note was missing in the originally published article. Therefore, the corrected version of the Acknowledgements’ section reads as follows:

This work was supported by the European Union's Horizon 2020 research and innovation program (grant agreement no. 848223). The authors thank Dr Nina Goldman for her critical revisions and language editing.

The correction will appear in the online version of the paper on the JMIR Publications website on September 7, 2022, together with the publication of this correction notice. Because this was made after submission to PubMed, PubMed Central, and other full-text repositories, the corrected article has also been resubmitted to those repositories.

