# Supplementary material for: Ethical Issues in Social Media Recruitment for Clinical Studies: Ethical Analysis and Framework
Source: J Med Internet Res. 2022 May 3;24(5):e31231. doi: 10.2196/31231 (PMC9115665; doi:10.2196/31231)
Supplement: Multimedia Appendix 1 [file jmir_v24i5e31231_app1.pdf]

Multimedia Appendix 1: Search algorithms for literature review.

**Challenges**	**challenges OR concerns OR problems AND social media AND recruitment AND clinical trial**
Stigmatization	stigmatization AND social media AND recruiting AND clinical trials
Social media algorithms	algorithms AND social media AND proprietary AND ethics
	algorithms AND social media AND inference AND recruitment
	algorithms AND social media AND categorization AND recruitment
Research ethics	research ethics AND social media AND recruitments AND clinical trials
Digital divide	Age AND social media
	Digital divide AND social media
	Socioeconomics status AND social media
Informed consent	Informed consent AND social media AND recruitment AND clinical trial
Data security	data AND security AND social media AND recruitment
	data AND security AND social media
**Benefits**	**benefits OR chance OR opportunities AND social media AND recruitment AND clinical trial**
Effectiveness	Effectiveness AND social media AND Recruitment
	Effectiveness AND social media AND Recruitment AND clinical trials
Hard-to-reach populations	social media AND recruitment AND hard-to-reach AND populations
	social media AND recruitment AND hard-to-reach AND populations AND clinical trials
Recruiting costs	social media AND reccruiment AND recruiting costs
Sample Diversity	social media AND recruitment AND bias
	social media AND recruitment AND diversity
	social media AND recruitment AND age
**Other**	
Demographics of social media plattforms	Demographics AND Facebook
	Demographics AND Instagram
Privacy (concept research)	privacy AND review
	privacy AND concept
	privacy AND social media
	information privacy
	information privacy AND social media
	data privacy AND social media
	data privacy AND social media
social media targeting algorithms	social media AND targeting AND recruiting AND ethics
	social media AND targeting AND recruiting AND clinical trials
	social media AND targeting AND ethics
	social media AND targeting AND recruitment
(ethics for) social media-based recruitment	social media AND recruitment AND ethics
	social media AND recruitment AND ethics AND clinicial trials
multi plattform approach for social media recruitment for clinical studies	plattforms AND recruitment AND social media

	plattforms AND recruitment AND social media AND clinicial trials
	multi-plattform AND recruitment AND social media
	multi-plattform AND recruitment AND social media AND clinical trials
	multi AND plattform AND recruitment AND social media
	multi AND plattform AND recruitment AND social media AND clinical trials
target group definition for social media recruitment for clinical studies	target AND group AND recruitment AND social media
	target-group AND recruitment AND social media
	target AND group AND recruitment AND social media AND clinical trial
	target-group AND recruitment AND social media AND clinical trial
