# Supplementary material for: Ethical Issues in Social Media Recruitment for Clinical Studies: Ethical Analysis and Framework
Source: J Med Internet Res. 2022 May 3;24(5):e31231. doi: 10.2196/31231 (PMC9115665; doi:10.2196/31231)

Multimedia Appendix 2. Checklist for researchers considering social media recruitment for clinical studies.

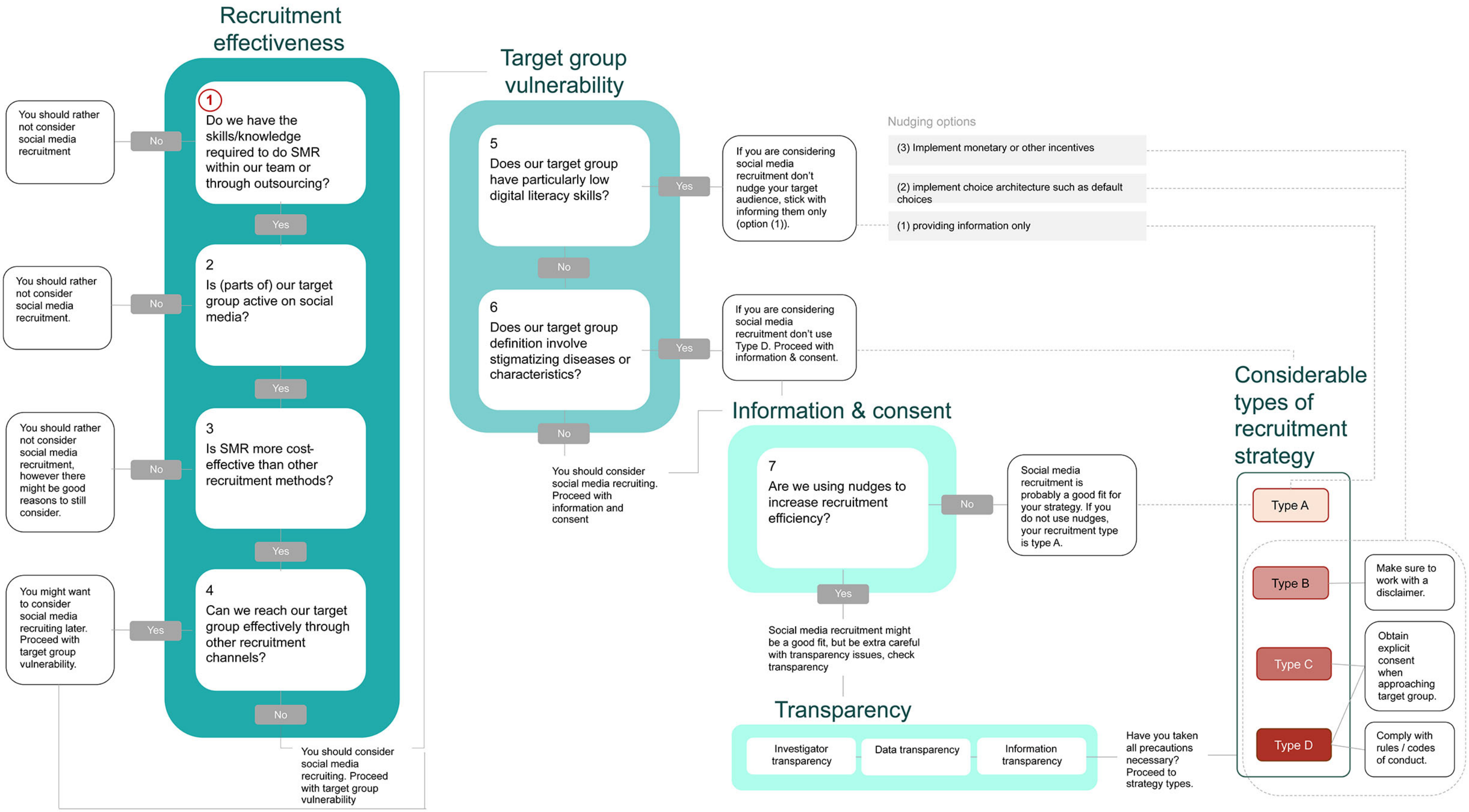

Supplement: Multimedia Appendix 2 [file jmir_v24i5e31231_app2.pdf]
